# A Case of Multibacillary Borderline Lepromatous Leprosy in the United States Treated With Alternative Therapy

**DOI:** 10.7759/cureus.66275

**Published:** 2024-08-06

**Authors:** Nicholas Chin, Logan R Smith, Sudeep Gaudi, Brooke Baldwin

**Affiliations:** 1 Dermatology, University of South Florida Morsani College of Medicine, Tampa, USA; 2 Dermatopathology, James A. Haley Veterans' Hospital, Tampa, USA; 3 Dermatology, James A. Haley Veterans’ Hospital, Tampa, USA

**Keywords:** borderline lepromatous, multibacillary, mycobacterium leprae, hansen's disease, leprosy

## Abstract

Hansen’s disease (leprosy) is a rare infectious disease with less than 300 recorded cases in the United States every year. In this report, we present an 80-year-old White male with a three-year history of slowly progressive, pink, well-demarcated, and annular patches, plaques, and papules that started on his thighs and spread to his abdomen, chest, back, and upper extremities. After sequence identification on biopsies confirmed the diagnosis of leprosy, the patient was treated with an alternative regimen of monthly moxifloxacin, rifabutin, and minocycline. After a month of treatment, the patient reported a reduction in his rash. This report highlights a case of multibacillary leprosy in the southeastern United States. It is a very interesting study as the selection of this patient’s treatment was complex, and he ultimately received an alternative therapeutic regimen. This case also highlights the rising incidence of leprosy in this region of the United States and stresses the importance of monitoring for warning signs indicative of this diagnosis to facilitate early treatment and attenuation of this disease.

## Introduction

Hansen’s disease (leprosy) is a disease caused by an infection with the acid-fast bacilli (AFB) *Mycobacterium leprae* or *Mycobacterium lepromatosis*. *M. leprae* is an obligate intracellular organism that is transmitted via respiratory droplets but rarely may be transmitted through contact with the skin. Leprosy is transmitted primarily through human-to-human contact and, interestingly, contact with the nine-banded armadillo, who serve as a natural reservoir for *M. leprae* [1-2]. This pathogen mainly targets Schwann cells in peripheral nerve fibers and macrophages in the skin, therefore presenting with neurological and dermatological symptoms [1]. The classic clinical presentation of leprosy is one or several hypopigmented or erythematous patches that have variable loss of sensation [3].

The diagnosis of leprosy is made with a combination of clinical symptoms and a skin biopsy and/or skin slit smear demonstrating AFB in tissues. The classic presentation of a patient with decreased sensation in a hypopigmented skin lesion should raise suspicion of leprosy. Polymerase chain reaction (PCR) is also often used to confirm the diagnosis of leprosy following biopsy [4-5]. Once diagnosed, leprosy can be classified by two different systems: one according to the World Health Organization (WHO) and one known as the Ridley-Jopling system. The WHO classification is simpler, with patients classified as paucibacillary (PB) if they have one to five skin lesions and a negative skin smear test and multibacillary (MB) if they have more than five skin lesions or a positive skin smear test [5]. The Ridley-Jopling system categorizes leprosy along a spectrum from tuberculoid leprosy (TT) to lepromatous leprosy (LL) based on a few clinical features, determined by the amount of host immune response to the infection. TT patients have a strong immune reaction, which prevents the systemic spread of the bacteria, while LL patients have a weak immune reaction, which allows the bacteria to spread widely. In between the two extremes, patients can also be classified as borderline tuberculoid (BT), midborderline (BB), and borderline lepromatous (BL) based on their degree of immune reaction, each classification having its own clinical features [5-6]. The current standard-of-care treatment for leprosy differs based on the type of leprosy (TT versus LL) and the various published guidelines. For instance, the National Hansen’s Disease Program (NHDP) has a more intense antimicrobial regimen, recommending dapsone and rifampin for 12 months for TT and dapsone, rifampin, and clofazimine for 24 months for LL. The WHO recommends a multidrug regimen of dapsone, rifampin, and clofazimine for six and 12 months for TT and LL, respectively [7-9]. Besides the standard regimen, both the WHO and the NHDP give alternative courses that are recommended in case of adverse drug reactions or lack of clinical response to therapy [8-9]. However, there has been an increasing use of alternative regimens as a primary treatment by clinicians.

In this case report, we present a patient with several pre-existing conditions in the southeastern United States with multibacillary, borderline lepromatous leprosy who received an alternative treatment course.

## Case presentation

An 80-year-old White male presented to our outpatient dermatology clinic with a three-year history of slowly progressive, pink, well-demarcated, and annular patches, plaques, and papules that started on his thighs and spread to his abdomen, chest, back, and upper extremities, as well as stocking and glove distribution paresthesia. He reported that the skin lesions are largely asymptomatic, denying pain or pruritus of the lesions. He also denied any systemic symptoms, including cough, shortness of breath, palpitations, abdominal pain, and muscle weakness. He notably denied any known contact with people with similar symptoms or any direct contact with armadillos; however, he has reported seeing armadillos in his yard. Our patient has not traveled internationally in the past 20 years. He spends most of the year in the North Carolina mountains and a few months in the Tampa area. At his home clinic in North Carolina, he was originally diagnosed with erythema multiforme, but his skin rashes and paresthesia did not resolve, causing him to present to our clinic.

**Figure 1:**
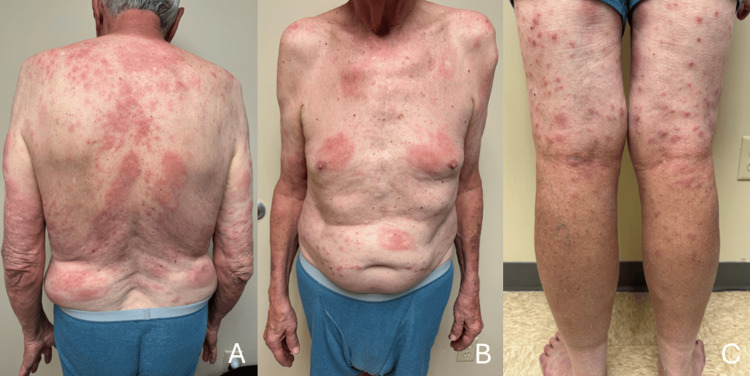
Clinical images of the patient 1A: Erythematous, well-demarcated confluent plaques on the patient’s back. 1B: Lesion with a clear targetoid appearance on the patient’s abdomen and edematous bilateral hands characteristic of lepromatous leprosy. 1C: Numerous scattered erythematous papules on patient’s bilateral posterior lower extremities.

**Figure 2:**
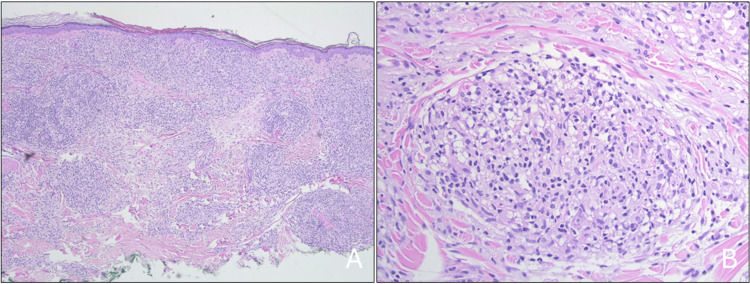
Histopathology slides 2A: Low-power examination reveals an exuberant infiltrate in the dermis (hematoxylin and eosin; 40X). 2B: Intermediate-power examination reveals the infiltrate to be granulomatous in nature (hematoxylin and eosin; 200X).

A physical exam revealed extensive indurated confluent plaques, papules, and targetoid lesions involving the trunk and lower extremities. In addition, the patient had erythematous patches on the back, abdomen, and arms bilaterally, with several lesions having a targetoid appearance (Figure 1). The physical exam also revealed swelling of the nose, bilateral hands, feet, cheeks, and ears. No nasal perforation or involution was present. He had diminished sensation in his bilateral hands, feet, and lower legs with no foot drop or motor deficits. No facial nerve palsies were apparent. Microscopic examination of shave biopsy specimens of the right arm and left leg revealed an exuberant granulomatous inflammatory infiltrate in the dermis (Figures 1, 2).

**Figure 3:**
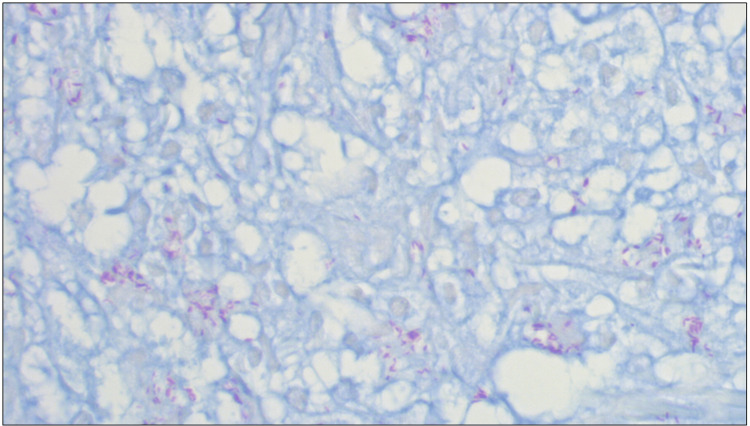
Fite stain Numerous acid-fast bacilli (red) are seen within the granulomatous infiltrate (Fite; 600X).

Special stains showed innumerable AFB- and Fite-positive bacilli within the granulomatous infiltrate (Figure 3). Sequence identification performed on paraffin-embedded tissue blocks revealed these organisms to be *M. leprae*, confirming the diagnosis of leprosy. Due to the extensivity and severity of skin lesions, the density of AFB in slit-skin smears, the presence of swelling in the face and distal extremities, and lack of any definitive lepromatous leprosy features, the patient was classified as having multibacillary, borderline lepromatous leprosy.

Due to the patient’s history of alcohol use, aortic and mitral valve bioprostheses, and atrial fibrillation treated with long-term anticoagulants (apixaban), his treatment selection was complex, and the case was reported to the NHDP for advice. Per their recommendation, the patient was started on a course of monthly moxifloxacin, rifabutin, and minocycline for 12 months. The patient was also started on methotrexate weekly with daily folic acid supplementation to mitigate the anticipated inflammatory response from the host immune system. After the first month of treatment, the patient reported fading of multiple raised lesions, as well as a general improvement in his neuropathy. He also reported that his swelling has improved.

## Discussion

While leprosy is not a common diagnosis in the United States, the prevalence of patients with leprosy has been rising over the past few years. According to the CDC, recorded leprosy cases have remained around 200 over the past decade. There was a transient decrease in recorded cases corresponding to the lockdown period, dropping from 216 in 2019 to 124 in 2021, but cases of leprosy in 2023 represent the highest number in the past decade, coming in at 225 [10]. The decrease from 2020 to 2022 is likely due to a combination of decreased transmission between people and decreased presentation to clinics. Nevertheless, the upsurge in recorded leprosy cases is significant. The southeast United States, particularly Florida, has the highest fraction of reported cases, with some papers published by the CDC even discussing leprosy being endemic to Florida due to the presence of nine-banded armadillos, specifically the Central Florida region, which accounts for 81% of the cases in Florida [10-11]. This makes it very important for clinicians in this region of the United States to keep leprosy as a differential, as early diagnosis and treatment of this disease are crucial for preventing further transmission and disability [12].

The exact mechanism of infection in this case is unclear. The patient denied direct contact with armadillos, had not traveled internationally in 20 years, and had no known contact with anyone with leprosy symptoms. However, the patient did report seeing armadillos in his backyard. In the southeastern United States, roughly 16.4% of armadillos carry the AFB *M. leprae *according to one study [13]. This, combined with the patient’s report that his dog was an “avid hunter,” provides a plausible explanation for his route of infection, if, for example, his dog contacted armadillo-contaminated soil and transported the soil on its fur to the patient. Given that this theory cannot be investigated, our patient’s mechanism of infection remains unknown.

This patient was administered a newer treatment regimen recommended by a physician at the NHDP. The traditional first-line treatment of leprosy has been a multidrug therapy (MDT) that consists of daily doses of dapsone, rifampin, and clofazimine [8]. Although MDT is highly effective, showing a 99% cure rate across different studies, there are many side effects that may present with this treatment [14]. There have been recorded cases of adverse or hypersensitivity reactions to dapsone, with studies even suggesting removing dapsone from the first-line multidrug therapy [14]. Clofazimine has additional risks, with case studies and reviews reporting reversible yet long-lasting clofazimine-induced pigmentation that localizes on the face and extremities [15-16]. On top of the adverse events that may occur with dapsone and clofazimine treatment, the high pill burden of MDT can result in lower patient adherence and quality of life [16]. Thus, there have been many new drug combinations that have been suggested for the treatment of leprosy. Although not yet outlined in official leprosy treatment guidelines, the NHDP has been recommending a treatment course consisting of monthly doses of rifampin, moxifloxacin, and minocycline [16-17]. This alternative regimen is only officially outlined by the WHO and NHDP guidelines for use if MDT fails or if the patient experiences an adverse drug reaction; however, more clinicians are beginning to select this regimen with great success. Although the literature is not yet strong enough to support this combination becoming the first-line treatment of leprosy, studies have shown that it has equal efficacy, with a lower risk of adverse effects [16,18]. In addition, the pill burden on the patient is drastically decreased. This report lends to the growing literature showing the efficacy of this drug regimen and highlights its potential as a new standard of care treatment for leprosy.

This patient also had a treatment course that was complicated by his long-term history of alcohol use and anticoagulants. Rifamycins (i.e., rifampin and rifabutin) are one of the most efficacious classes of antibiotics in treating leprosy; this patient was taking rifabutin monthly. However, the efficacy of rifamycin also comes with several side effects and contraindications. Rifamycins can result in higher liver function tests, reflecting increased strain on the liver when administered [9]. Rifamycin can therefore enhance the toxicity of alcohol, and as a long-time alcohol user, the patient was warned against the consumption of any alcohol while undergoing his drug regimen. In addition, due to his atrial fibrillation, the patient was on daily apixaban, a direct factor Xa inhibitor that has better efficacy and lower mortality compared to warfarin [19]. However, rifamycins have a significant drug interaction with direct oral anticoagulants. A systematic review found that the bioavailability of oral apixaban was decreased by 54% following administration of rifamycin [20]. This puts patients who are on both anticoagulants and rifamycin at high risk for a thromboembolic event. In the end, it was decided that the patient would remain on apixaban with close monitoring. Future research should focus on whether increasing the dose of apixaban after concurrent usage of rifamycin is recommended in reducing stroke risk.

## Conclusions

Leprosy, although still a rare disease, is a growing concern in the southeast region of the United States. This report adds to the growing literature about various cases of leprosy arising in Florida specifically. The alternative regimen used by this patient, with his treatment selection complicated by comorbidities and the uncertain mechanism of infection, makes it a unique case. Although his symptoms were extensive, the patient is showing great improvement in his symptoms after two months of his alternative regimen, which highlights the utility of using second-line therapeutics for the treatment of leprosy. This case should also underscore the increasing incidence of leprosy in this region and stress the importance of monitoring for certain alarm signs that would point to this diagnosis to facilitate early treatment and attenuation of this disease.
